# Yoga for the Management of Attention-Deficit/Hyperactivity Disorder

**DOI:** 10.7759/cureus.20466

**Published:** 2021-12-16

**Authors:** Luxhman Gunaseelan, Manasvi S Vanama, Farwa Abdi, Aljeena Qureshi, Ayesha Siddiqua, Muhammad A Hamid

**Affiliations:** 1 Medicine, Saba University School of Medicine, Toronto, CAN; 2 Health Sciences, University of Waterloo, Toronto, CAN; 3 Health, York University, Toronto, CAN; 4 Health Sciences, McMaster University, Toronto, CAN; 5 Internal Medicine, Dow University of Health Sciences, Dow International Medical College, Karachi, PAK; 6 Pediatrics, University of Toronto, Toronto, CAN

**Keywords:** therapy, yoga, pediatric, mind–body, mindfulness, meditation, attention-deficit/hyperactivity disorder (adhd), alternative

## Abstract

Yoga has been shown to play a role in reducing the symptoms associated with the inattentive and hyperactive-impulsive forms of attention-deficit/hyperactivity disorder (ADHD). The medical history and clinical findings for a nine-year-old patient presenting with difficulty paying attention and impulsive speech and actions at home and school are presented. After the diagnosis of combination type ADHD by assessment of DSM-5 criteria, both at home and school and through parent and teacher evaluations using National Institute for Children’s Health Quality (NICHQ) Vanderbilt Assessment Scales, the patient initiated a yoga training regimen. Six months after initiating the yoga training regimen, follow-up parent and teacher questionnaires revealed improvement in both the inattentive and hyperactive-impulsive symptoms. Literature sourced from the PubMed database to explore the efficacy of yoga for ADHD was used to support the research hypothesis that a structured yoga training regimen improves the symptoms associated with the inattentive and hyperactive-impulsive forms of ADHD, and thus, yoga is recommended as a management technique for individuals with ADHD.

## Introduction

Attention-deficit/hyperactivity disorder (ADHD) is a neurological disorder characterized by difficulties in paying attention, excessive activity, or controlling behaviour, that is not appropriate for a person's age [1]. ADHD is diagnosed and evaluated using the DSM-V criteria, as well as questionnaires such as the National Institute of Children’s Health Quality (NICHQ) Vanderbilt Assessment Scale that is filled out by Parents and Teachers [2]. The symptoms of ADHD are generally managed through pharmacotherapy and/or behavioural therapy. Although pharmacotherapy such as stimulant medications can be effective in the management of ADHD symptoms, there are potential side effects, which can influence parents’ decision to opt for alternative treatments [3]. Furthermore, it is unclear whether behavioural therapies that target behaviours in specific environments can be effectively used in different situations [3].

ADHD is usually diagnosed in about 3% to 7% of school-age children [3]. In the 2016 U.S. National Survey of Children’s Health (NSCH), it was estimated that a total of 6.1 million children between the ages of two and 17 had received an ADHD diagnosis [4]. It is important to note that this number only does not reflect the unknown number of children who go undiagnosed every year. Furthermore, not all of the children who are diagnosed with ADHD can receive treatment due to various reasons such as socioeconomic status, or refusal of pharmacotherapy [4]. In light of the recent COVID-19 pandemic, changes to a child’s routine and access to formal and informal supports can have significant adverse impacts on a child with neurodevelopmental conditions such as ADHD. An increase in screen time, decrease in physical activity, and changes in sleep and eating patterns were found to lead to worsening symptoms and health, as well as increased levels of stress [5]. With video conferencing technologies such as Zoom, online yoga classes could be quite beneficial and feasible for individuals with ADHD, to manage their symptoms during the pandemic. 

Yoga therapy

Yoga is a mind-body therapy that can benefit health by regulating attention, decreasing psychological stress, and self-regulation [6]. Through the use of physical postures (asanas), breathing exercises (pranayama), and meditation techniques, individuals can improve their musculoskeletal, cardiovascular, and nervous system functions, which can affect their emotional well-being. Yoga has been shown to improve several physiological functions and improve cognitive domains such as executive functions, attention, intelligence, memory and concentration. Yoga can help reduce symptoms of various illnesses such as irritable bowel syndrome, chronic pain, neurodegenerative conditions, and depression and post-traumatic stress disorder (PTSD) [6]. It is important to note that psychological conditions such as depression are common comorbid conditions with ADHD, suggesting the potential benefits of yoga for the overall well-being of the individual [3]. Yoga helps individuals with ADHD by reducing hyperactivity and distractibility, which improves cognitive function and academic performance. Pranayama, or yogic breathing techniques, are especially effective in reducing hyperactivity and distractibility in ADHD patients, allowing them to calm down and follow instructions. There is also evidence that vagal control during meditation correlates with differential activation in brain regions regulating threat appraisal, interoception, emotion regulation, and facilitating greater flexibility in response to challenge [6]. Pranayama is also very simple to learn and perform, thus being excellent for ADHD patients starting yoga therapy. More complex yogic movements will then be taught once ADHD patients can master pranayama. Parents can also do yoga with their children as a family treatment approach, in the form of Sahaja yoga meditation, which will allow parents to relieve their stress, help manage their child’s behaviour, and also improve the child-parent relationship [5].

## Case presentation

We present the case of a nine-year-old South Asian male who was seen with his parents for the evaluation of a two-year history of experiencing symptoms associated with ADHD. This individual was recruited from a yoga centre in the Greater Toronto Area and was not on any other interventions to manage his ADHD symptoms, at the time of the yoga intervention. His parents and school teacher observed frequent instances of fidgeting, difficulty remaining seated during mealtime and class, frequent talking and interruptions, and difficulty waiting turns. They also observed declining school performance over two years, frequent careless mistakes in schoolwork, difficulty completing homework and chores, and frequently misplacing toys and schoolbooks. He had no past medical or psychiatric history such as central nervous system infections, seizures, recurrent otitis media, mood disorders, anxiety, or conduct disorder. Birth history revealed no perinatal infections or complications, or perinatal exposure to tobacco, drugs or alcohol. Medication history for both prescribed or over-the-counter drugs was negative. In terms of family history, his father also struggled with ADHD as well as major depressive disorder. A physical examination revealed a healing superficial abrasion of the left knee from a recent fall. Further cardiac examination revealed a regular rate and rhythm, no murmurs, rubs, gallops or other cardiac abnormalities. A neuropsychiatric exam revealed a person who was oriented to person, place and time, with an agitated demeanour and poor eye contact. He spoke at a fast rate, and his mood was nervous, while his speech and thought process were coherent, and no auditory or visual hallucinations were noted. 

NICHQ Vanderbilt Assessment Scale assessments were collected from both the patient’s parents and teacher. The initial parent questionnaire revealed that the child’s total symptom score was 45 and the average performance score was 4.5. In the teacher’s assessment of the child, the child scored 44 on the total symptom score and 4.25 on the average performance score. The results of these questionnaires were indicative of the diagnosis of ADHD. The parents refused any pharmacologic therapy for their child’s ADHD, instead opting to enrol their child in a daily, online evening yoga program conducted through Skype, where the child performed a set of asanas that are outlined in Table 1. There were three different programs of one hour in length that incorporated various sets of asanas and were alternately practiced on a daily basis, under parental supervision. In general, the daily yoga routine consisted of a 10-minute meditation, followed by 40 minutes of asanas, and finally 10 minutes of Pranayama breathing techniques at the end of the class. The sets of asanas in each program were not followed in any particular order.

**Table 1:** Summary of daily yoga intervention for the management of ADHD.

Program Sets	Meditation (10 minutes)	Asanas performed (40 minutes)	Pranayama (10 minutes)
1		Eagle, Frog, Fish, Crocodile, Tortoise, Dragonfly, Camel, Lion, and end with Child’s Pose	Breathing techniques: Kapalbathi, Anuloma Viloma, Bhastrika
2		Tree pose, Scorpion, Blue Whale, Pigeon, Tortoise, Lion, Cobra, Corpse Pose.	
3		Eagle, Tree, Scorpion, Fish, Pigeon, Crocodile, Dragonfly, Cobra, Child's pose	

Each asana can help with either concentration, aggression, anxiety or self-esteem [1]. Furthermore, they have many benefits that seem to help manage the symptoms of ADHD such as balance, focus, calming the mind, boosting confidence, helping with emotional stability, and more [1]. The benefits of each asana are described in Table 2 [1].

**Table 2:** The benefits of each asana

Characteristic	Asana/Posture	Benefits
Concentration	The Eagle	Balances posture, develops concentration and focus, nurtures determination and inner conviction, improves attention span, calms the mind, improves the eye muscles
	The Frog	Gives a quick boost of energy, helps “let off some steam”
	The Child’s Pose	Settles the learner when feeling hyperactive or overtired, restores energy, calms the mind, and can help induce sleep
	Tree Pose	Standing poses where concentration is needed to stand completely
	The Scorpion	Aids concentration and balance, boosts confidence
Aggression	The Fish	Improves posture and chases away negative feelings
	The Crocodile	Strengthens the back and gives energy, helps to release anger and aggression
	The Blue Whale	Provides a little “lift”, calms one down
	The Pigeon	Helps to calm an agitated mind
Anxiety	The Tortoise	Helps one imagine being protected by a strong shell, helps one feel safe and quiet
	The Dragonfly	Helps to develop patience and emotional stability, induces feelings of emotional calm and quiet before going to bed
	The Corpse Pose	A deep relaxation exercise that can keep the learner relaxed and centred
Self-Esteem	The Camel	Helps to correct poor or lazy posture, helps all learners to stand tall and feel proud of who they are
	The lion	Energizes the body and mind, builds self-confidence and improve communication skills, helps with anxiety
	The Cobra	Keeps the spine supple and healthy, tones the nerves to improve communication between the brain and body, helps the learner feel strong and powerful

At the six-month follow-up after initiating the yoga training regimen, the parents noted subjective improvement in their child’s ADHD symptoms. His parents and school teachers observed decreased instances of fidgeting, improved ability to remain seated during mealtime and during class, improved ability to complete homework and chores and improved school performance. The follow-up teacher questionnaire which was obtained after six months revealed a total symptom score of 27/54 and the average performance score for questions was 3.25 (Tables 3, 4). The parent follow-up questionnaire at the six-month visit revealed a total symptom score of 35/54 and the average performance score was 3.6, indicating an improvement from the initial score of 4.25 (Tables 5, 6).

**Table 3:** Summary of NICHQ Vanderbilt Assessment scored by the participant’s teacher at baseline. NICHQ - National Institute for Children’s Health Quality

NICHQ Vanderbilt Assessment Scale Summary - Teacher Informant	Teacher Score
Total number of questions scores 2 or 3 in questions 1-9	8
Total number of questions scores 2 or 3 in questions 10-18	9
Total symptom score for questions 1-18	44
Total number of questions scores 2 or 3 in questions 19-28	0
Total number of questions scores 2 or 3 in questions 29-35	0
Total number of questions scores 2 or 3 in questions 36-43	7
Average Performance Score	4.25

**Table 4:** Summary of NICHQ Vanderbilt Follow-up Assessment scored by the participant’s teacher. NICHQ - National Institute for Children’s Health Quality

NICHQ Vanderbilt Assessment Scale Summary - Teacher Informant- Follow Up	Teacher Score
Total symptom score for questions 1-18	27
Average Performance Score	3.25

**Table 5:** Summary of NICHQ Vanderbilt Assessment scored by the participant’s parents at baseline. NICHQ - National Institute for Children’s Health Quality

NICHQ Vanderbilt Assessment Scale Summary - Parent Informant	Parent Score
Total number of questions scored 2 or 3 in questions 1-9	9
Total number of questions scores 2 or 3 in questions 10-18	9
Total symptom score for questions 1-18	45
Total number of questions scores 2 or 3 in questions 19-26	0
Total number of questions scores 2 or 3 in questions 27-40	0
Total number of questions scores 2 or 3 in questions 41-47	0
Total number of questions scores 4 or 5 in questions 48-55	7
Average Performance Score	4.5

**Table 6:** Summary of NICHQ Vanderbilt Follow-up Assessment scored by the participant’s parents. NICHQ - National Institute for Children’s Health Quality

NICHQ Vanderbilt Assessment Scale Summary - Parent Informant- Follow Up	Parent Score
Total symptom score for questions 1-18	35
Average Performance Score	3.6

## Discussion

To put our case in context, a literature search on PubMed was used to identify five studies that investigated yoga as an alternative or complementary therapy for the treatment of ADHD. A total of 145 study participants, across five studies, were considered. All studies were performed in the paediatric population, ranging from ages three to 16.

A review paper by Mehta et al. investigated the efficacy of yoga on improving ADHD symptoms as a low-cost approach to ADHD that is useful to treat children in developing countries [7]. Research demonstrated that ADHD is prevalent in all areas of the world, and yet treatment for children in developing countries remains a challenge. In this study, a program that combined yoga meditation and multimodal behavioural therapy was developed to target children with ADHD aged six to 11. The program was low-cost as it hired trained high school volunteers and integrated the program within the public school [7]. The severity of the participants’ ADHD was represented by a performance impairment score. Improvement in ADHD was assessed by the proper performance of each asana, academic performance, as well as a subjective review of the children’s behaviour by their parents and teachers, compared to the performance impairment score at baseline, before yogic therapy. Three healthy subjects without ADHD were also included in the study, to serve as the control group. After 6 weeks in the program, 90.5% of children showed improvement as measured by their performance improvement scores. Parent and Teacher evaluations of behaviour exemplified an improvement in 45/49 students in the study in December 2007 from the baseline in May 2007 [7]. This study demonstrated that yoga and meditation can cost-effectively manage ADHD and that children could successfully learn both yoga and meditation from high school students, irrespective of their age, or ADHD type [7].

Another study, conducted by Beart and Lessing explored the impact of yoga on the concentration and behaviour of 10 attention deficit hyperactivity disorder (ADHD) patients, using parent and teacher assessments [1]. The use of yoga to reduce typical ADHD behaviour such as aggression, anxiety, and low self-esteem, was examined in this study. Semi-structured interviews with parents, teachers and ADHD patients were conducted to assess the level of typical ADHD behaviour in each patient, before and after the yogic treatment. All patients were children aged 9 or 10 years old, who were diagnosed with ADHD in preschool. Eight out of ten patients were taking medication at the time of the study to manage their ADHD symptoms. Overall, the perception of parents and teachers was that yoga had a positive influence on the behaviour of most of the patients with ADHD, showing that there is a potential benefit in using yoga to support ADHD symptoms such as a lack of concentration, high levels of aggression and anxiety, and an improvement in self-esteem [1].

The next study, conducted by Harrison et al, investigated the efficacy of Sahaja yoga meditation on the management of ADHD in children [8]. Forty-eight children participated in the study, with the majority of children receiving medications such as Ritalin and dexamphetamine. Parents and children participated in regular meditation sessions twice weekly for a duration of six weeks. Treatment efficacy was assessed through parent ratings of children’s ADHD symptoms, self-esteem and child-parent relationship quality, before and after yoga treatment. This information was obtained through three sources: child self-report questionnaires, and Conners parent and teacher questionnaires [8]. Assessments were conducted at three checkpoints: at the commencement of the meditation program in the first week, during the third week of the program, and at the end of the program in the sixth week. ADHD symptom severity, as depicted by Conner Test scores decreased after yogic therapy, from 22.33 to 14.50 in the group taking no medication, and from 22.6 to 14.65 in the group receiving medication [8]. The self-report questionnaires show that the study participants felt an improvement in their behaviour, self-esteem and relationship quality. They described benefits at home such as improved sleep patterns and decreased anxiety, as well as improvements at school including improved concentration and reduced conflict. Additionally, parents reported feeling less stressed, as they were able to better manage their children’s behaviour [8]. This study demonstrated that yoga improved the symptoms of ADHD, whether or not it was used as a complementary treatment with medication. Furthermore, the shows that yoga can help manage symptoms with reduced dosages of medication that is already being taken. 

Varambally et al. examined yoga as a complementary therapy in children with moderate to severe ADHD [9]. The study was performed on children between five and 16 years of age diagnosed with moderate to severe ADHD. A total of nine children, eight males and one female, were recruited into the study and the participants were given yoga training daily until they were discharged. They were rated on Conners' rating scale, ADHD-rating scale-IV (ADHD - RS IV) and Clinical Global Impression (CGI)-Severity scales [9]. They were rated, at the beginning of the study, and the end of the first, second and third months after completion of the study. All but one of the children were on medication. The results of the study show that after yoga therapy, ADHD symptoms were reduced, in all rating standards. The greatest improvement was seen after the completion of the first month of the yogic therapy. During this time, many patients continued with their yogic exercises on their own. However, after the second and third months, symptom scores slightly increase as patients are less likely to be compliant long-term [9]. This shows that yoga should be done regularly to manage ADHD symptoms throughout an individual’s life and can serve as an effective complement to pharmacotherapy in ADHD.

In a more recent study, yoga was evaluated as front-line behavioural therapy for the management of ADHD symptoms in preschoolers aged three to five years old [10]. This mixed-methods randomized controlled trial evaluated participants using parent and teacher ratings on both the ADHD RS-IV Preschool version and the Strengths and Difficulties Questionnaire (SDQ). Additionally, attention was objectively measured by the Kinder Test of Attentional Performance (KiTAP), while heart rate variability (HRV) was used to measure the self-regulation of each participant. Twenty-three preschoolers participated in the six-week yoga intervention that was based at home and school, with 12 (group 1) of the participants completing the intervention in the first six weeks and 11 (group 2) completing the intervention from week 6 to week 12 [10]. The children were assessed using the measures described above at baseline at which point there were no significant differences between the groups, at the six-week point, the 12-week point, and three months after completion of the 12-week intervention period. The study concluded that there were modest improvements in the ADHD symptoms after analyzing the KiTAP scores for each group at each checkpoint identified above. Particularly, they noticed that the inattentive and combined symptoms markedly decreased after the practice of yoga [10]. In conclusion, the use of yoga as a technique to improve symptoms of ADHD was successful in this study. However, the completion of a study using measures such as KiTAP, but with a clear control group is recommended to provide stronger support and evidence for yoga in managing ADHD symptoms (Table 7).

**Table 7:** Summary of literature review.

Authors	Study Design	Participant Characteristics	Intervention	Results
Mehta et al., 2011	A school-based open-label exploratory study	n = 55 (Teacher assessments)	Duration: twice a week for 12 months.	Baseline Parent: median = 9, range = 4–20
		n=49/55 (Parent assessments)		Baseline Teacher: median = 13, range = 7–21
		Ages: 6-11		Teacher’s 6-week follow-up: median = 4, range = 1–9 (P < 0.0001 Wilcoxon signed rank test).
		ADHD types: 67.1% combined; 21.4% inattentive; 11.4% hyperactive/impulsive	Intervention: It is a peer-mediated interventional program consisting of yoga, meditation and play therapy maintained by trained high school student volunteers	Parent’s 5-week follow-up: median = 6, range 2–18 (P < 0.001 Wilcoxon signed rank test)
			Assessment methods: Parent and Teacher Vanderbilt Questionnaires were used	Parent’s 6-month follow up: median = 5, range = 0–18 (P < 0.001 Wilcoxon signed rank test)
				Teacher’s 1 year follow-up: median = 0.5, range = 0–14 (P < 0.0001 Wilcoxon signed rank test)
					Beart & Lessing, 2013	Exploratory study Qualitative semi-structured interviews	N = 10 n=8 taking Ritalin (6) or Concerta (2) And n=2 taking no medication	Duration: 6 weeks, twice a week for 40-minute sessions	Qualitative results: “Less aggressive”, “improved self-esteem”, “more confident”, “calmer behaviour”, “improved concentration” etc.
							Age: 9 years old (n=7) And 10 years old (n=3)	Assessment methods: Children’s Apperception Test (CAT), the Lawrence Self-Esteem Questionnaire (LAWSEQ), Parent and Teacher interviews	Overall: the yoga intervention appeared to have had a positive effect on all participants, to varying degrees.
Harrison et al., 2004	An open trial treatment program	N = 48	Duration: 6-week programme for twice-weekly 90 minute sessions	Baseline ADHD symptoms were moderately high, M = 22.65, and varied across the 48 participants (SD = 4.36; range: 15–30).
		n= 31 receiving medication	Intervention: Non-drug adjunctive intervention using Sahaja Yoga Meditation	Post treatment ADHD symptoms: SD = 4.91, range 0–19 (35% improvement) (t = 8.23, p < .001)
		n=14 receiving no medication	Assessment Methods: Biobehavioural Indicators of Self-Esteem questionnaire, An abbreviated version of Burnett’s (1994) 40-item self-evaluation and self-description measure, Peabody Picture Vocabulary Test – Third edition (PPVT-III), and child interviews	No medication (n=6) Mean score reduction after 6 weeks = 7.83, S.D. = 5.15
		n=3 unknown medical information		Medication (n=20) Mean score reduction after 6 weeks = 7.95, S.D. = 4.97
		Age: 4-12		Medication vs. no medication statistically not significant
				Reduced dosage (n=11) Mean score reduction after 6 weeks = 10.18, S.D. = 4.79
				No change of dosage (n=9) Mean score reduction after 6 weeks = 5.22, S.D. = 3.83
				Change in dosage vs. no change t = 2.51, p<0.02
Varambally et al., 2013	A hospital-based open-label exploratory study	N = 9	Duration: at least 8 days, six 1-hour sessions, monthly follow up for 3 months	The reduction was statistically significant for scores between baseline and discharge
		Age: 6-13	Intervention: The yoga program consisted of Sukṣmavyayāma (loosening exercises), Yogāsana (physical postures), Prāṇāyāma (breathing exercises) and meditation in the form of Nādānusandhāna (OM chanting)	P=0.014 on CARS
			Assessment Methods: ADHD rating scale-IV (ADHD-RS), Conners' abbreviated rating scale (CARS), and clinical global impression (CGI) Severity	P=0.021 on ADHD-RS
				P=0.004 on CGI
				There was no significant reduction in the scores during the follow-up. By the third month, scores were returning to baseline.
Cohen et al., 2018	Randomized waitlist-controlled trial	n = 23	Duration: Total 12 weeks (both groups)	At the 6 week follow up: Group 1 had faster reaction times on the KiTAP task (p = 0.01, 95% confidence interval [CI], 2371.1 to 259.1, d = 21.7) than Group 2.
				Group 1 had fewer distractibility errors of omission (p = 0.009, 95% CI, 214.2 to 22.3, d = 21.5) than Group 2
				Group 1 had more commission errors (p = 0.02, 95% CI, 1.4–14.8, d = 1.3) than Group 2
		Group 1: n=12 (practiced yoga first)	Intervention: home- and school-based children's yoga intervention.	Children in Group 1 with more severe symptoms at baseline showed improvement versus control on parent-rated Strengths and Difficulties Questionnaire (SDQ) hyperactivity-inattention (b=22.1, p = 0.04, 95% CI, 24.0 to 20.1)
				Children in Group 1 with more severe symptoms at baseline showed improvement versus control on inattention on the ADHD Rating Scale (b=24.4, p = 0.02, 95% CI, 27.9 to 20.9).
		Group 2: n=11 (practiced yoga second)	Assessment Methods: ADHD RS-IV Preschool Version18, Strengths and Difficulties Questionnaire19 (SDQ), Kinder Test of Attentional Performance (KiTAP), and heart rate variability (HRV) as a physiologic index of self-regulation.	HRV measures did not differ between groups.
		Age: 3-5		

## Conclusions

In conclusion, it is recommended that yoga be used as a complementary therapy to medication so that doses can be lowered or eliminated. Our case study exemplified the effectiveness of using yoga as a standalone treatment for ADHD, as improved ADHD and performance scores were observed in the individual. Further research should be conducted on the effectiveness of each asana on ADHD to create an ideal yoga routine for individuals to use to manage their symptoms. Studies with a larger number of participants should also be conducted to compare the effectiveness of yoga in medicated and unmedicated ADHD patients. More importantly, research on ADHD should continue to be done as we still do not have a clear understanding of the nature of the disease. Furthermore, with mental health on the rise as a result of the pandemic, yoga can be further explored to analyse the benefits it has in reducing stress and improving focus and concentration in a remote learning and working environment.

## Figures and Tables

**Figure 1:**
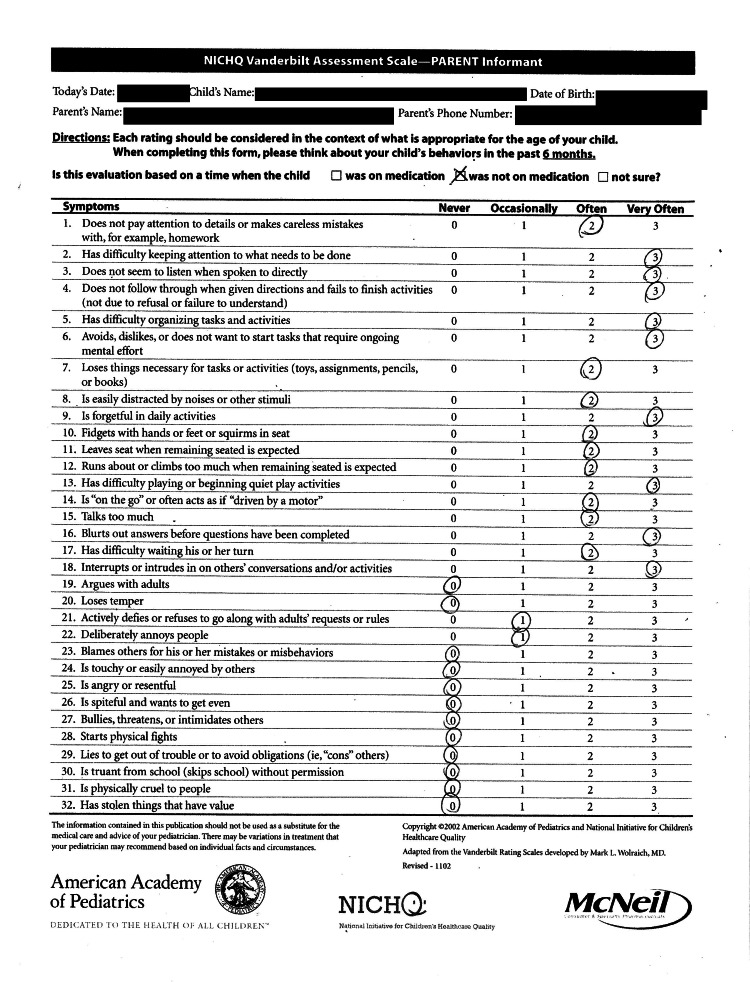
NICHQ Page 1: Parent assessment at baseline

**Figure 2:**
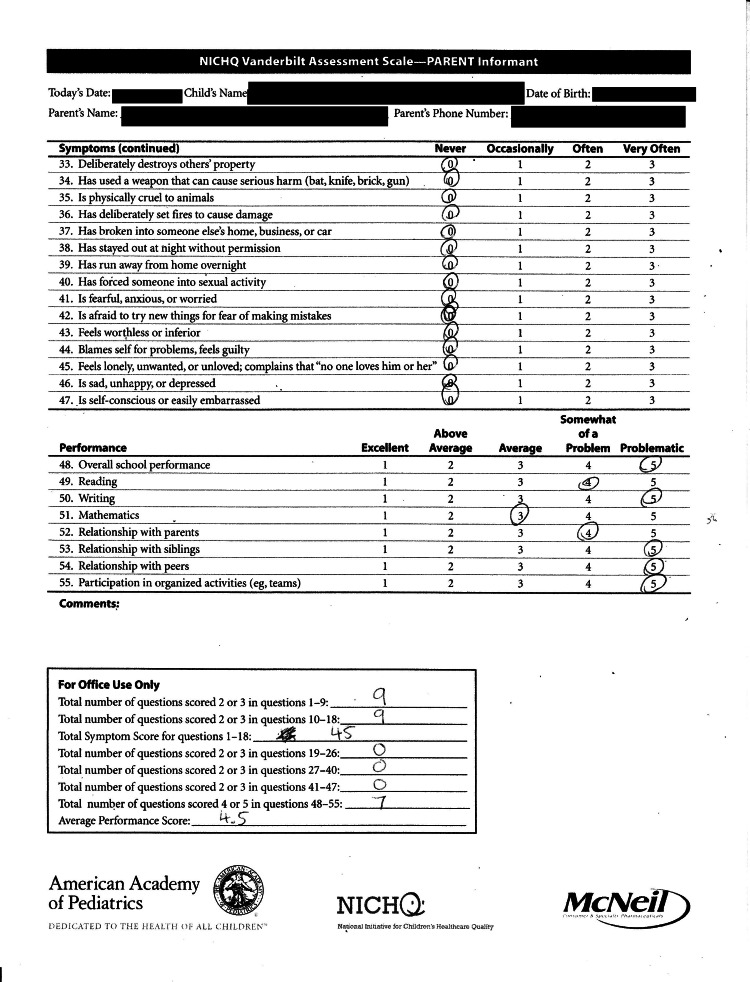
NICHQ Page 2: Parent assessment at baseline continued

**Figure 3:**
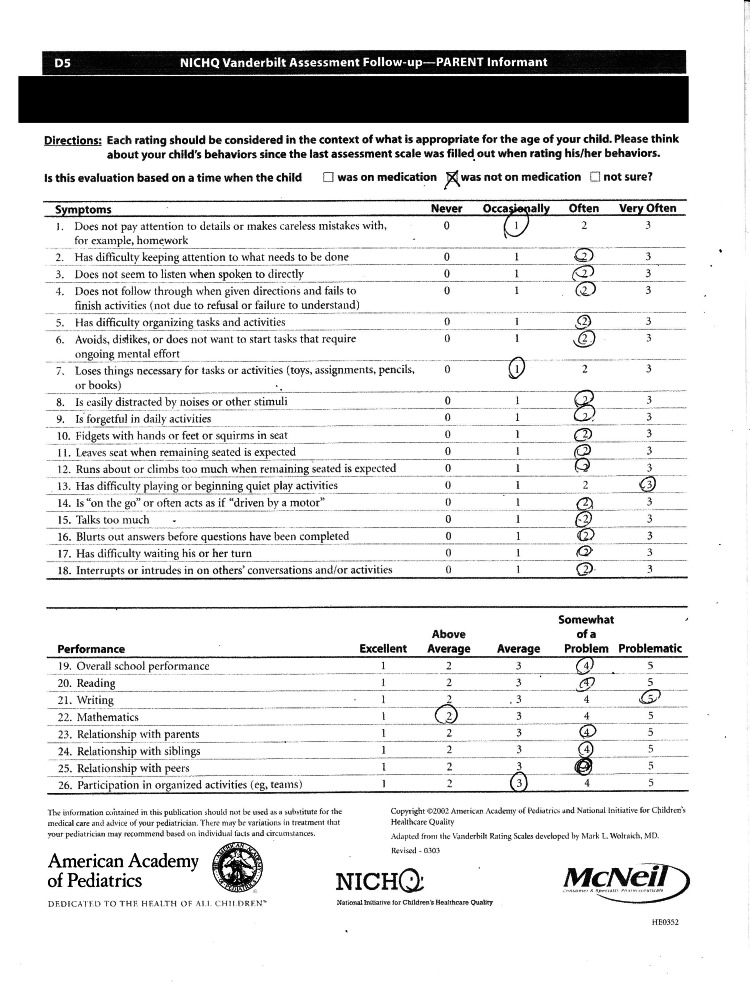
NICHQ Page 3: Parent assessment at follow-up

**Figure 4:**
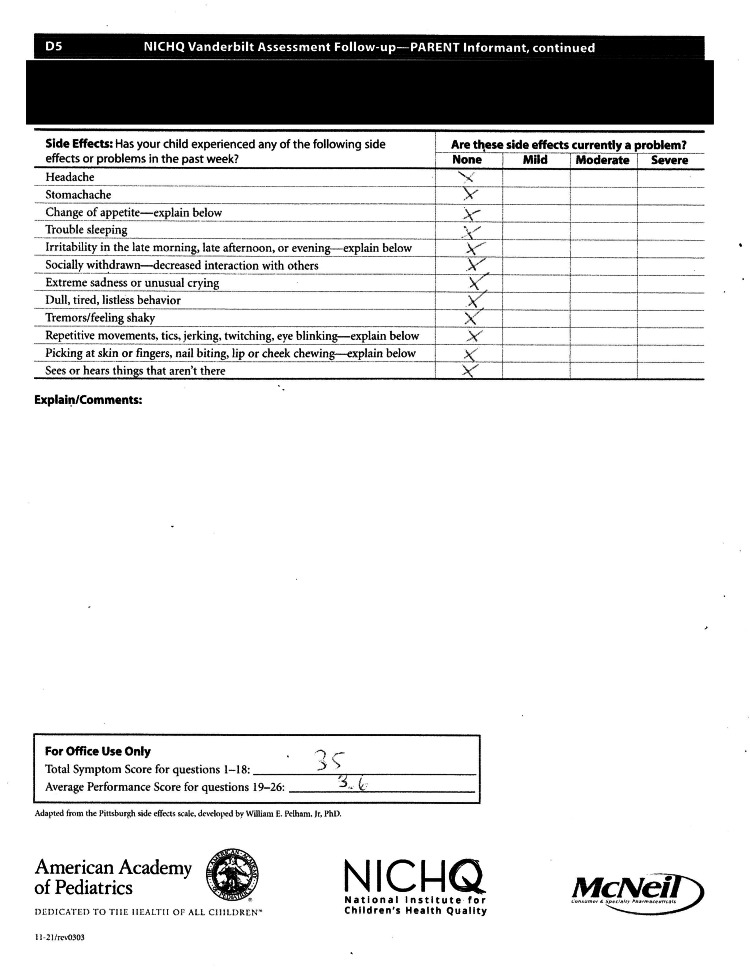
NICHQ Page 4: Parent assessment at follow-up continued

**Figure 5:**
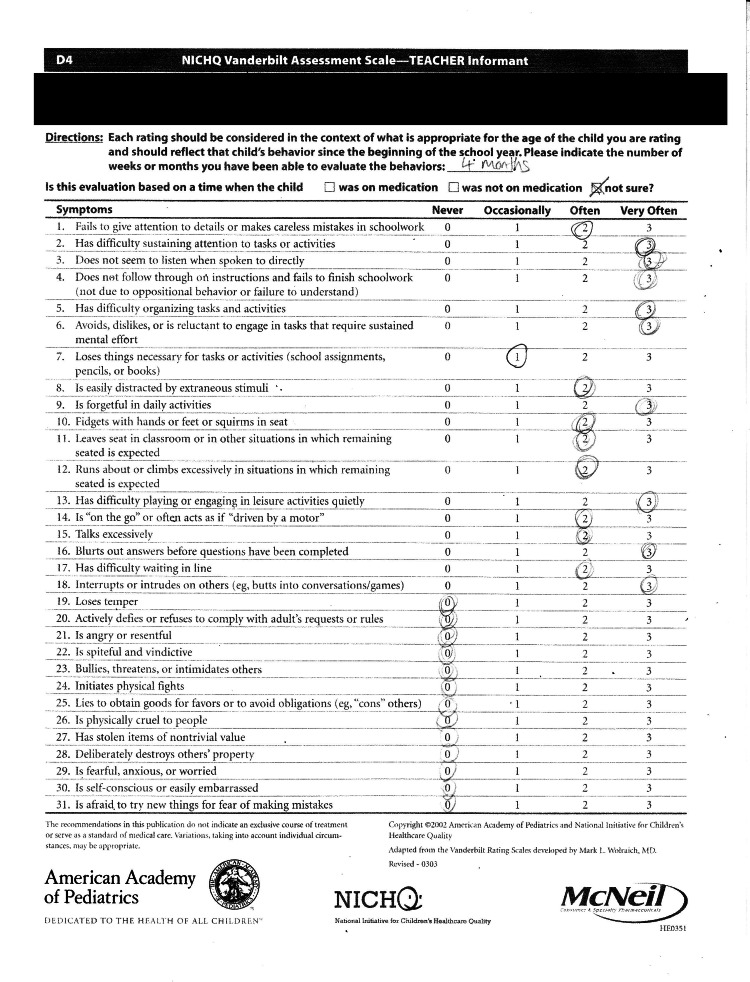
NICHQ Page 5: Teacher assessment at baseline

**Figure 6:**
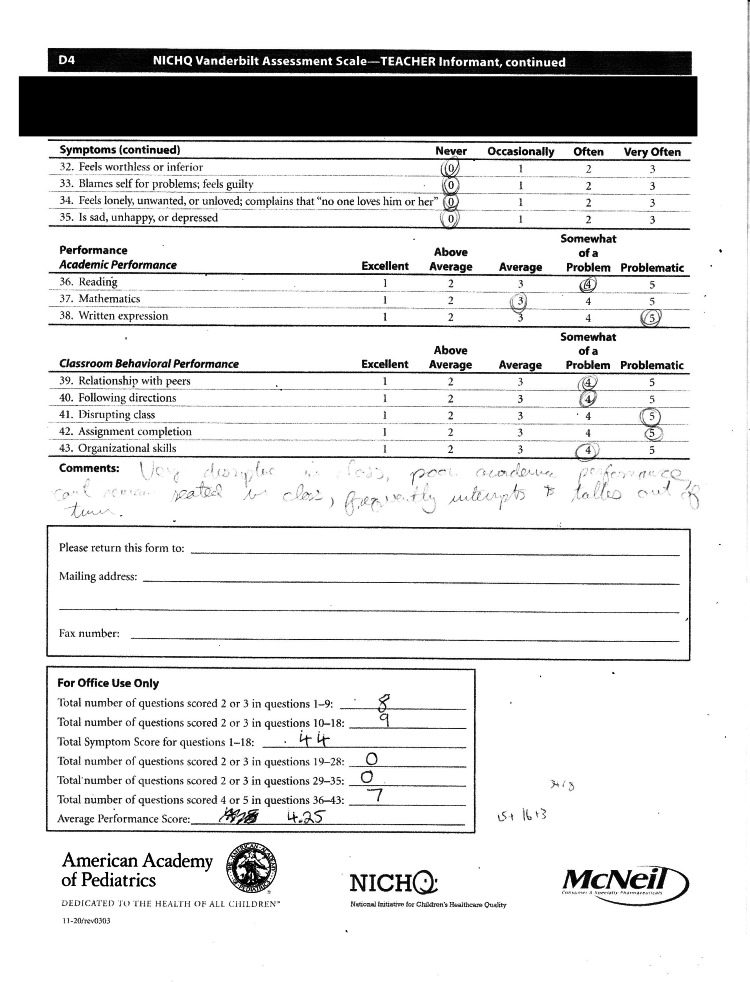
NICHQ Page 6: Teacher assessment at baseline continued

**Figure 7:**
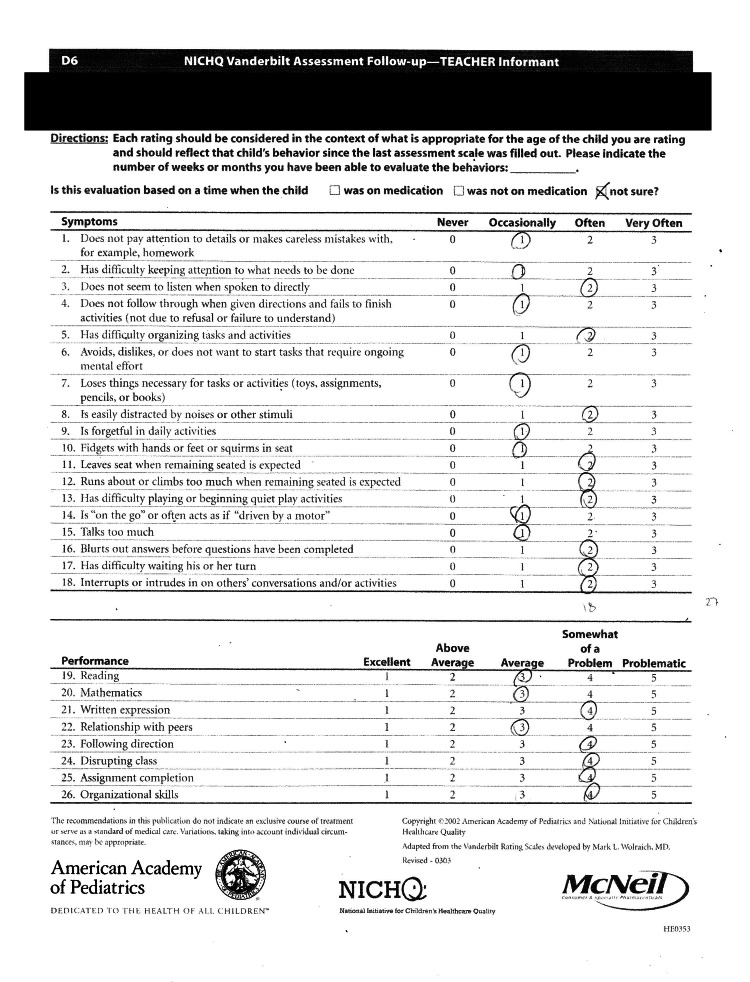
NICHQ Page 7: Teacher assessment at follow-up

**Figure 8:**
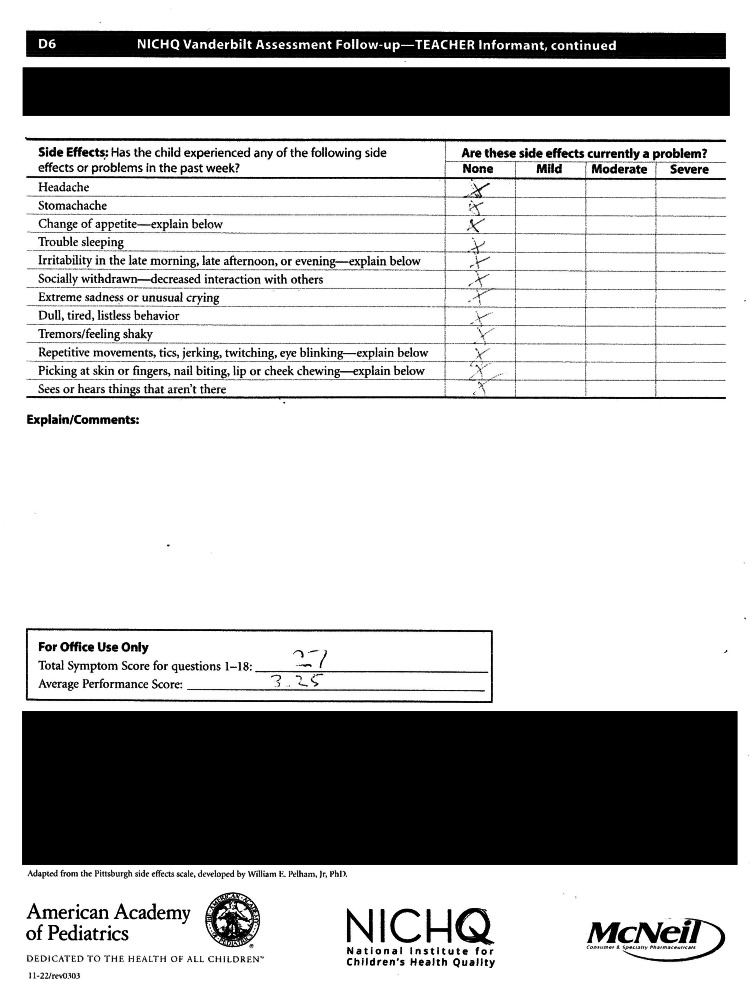
NICHQ Page 8: Teacher assessment at follow-up continued
